# Psychosomatic syndromes and anorexia nervosa

**DOI:** 10.1186/1471-244X-13-14

**Published:** 2013-01-09

**Authors:** Giovanni Abbate-Daga, Nadia Delsedime, Barbara Nicotra, Cristina Giovannone, Enrica Marzola, Federico Amianto, Secondo Fassino

**Affiliations:** 1 Department of Neuroscience, Section of Psychiatry, Eating Disorders Centre, University of Turin, Turin, Italy

**Keywords:** Anorexia nervosa, Eating disorders, Psychosomatic syndromes, Illness denial, Alexithymia

## Abstract

**Background:**

In spite of the role of some psychosomatic factors as alexithymia, mood intolerance, and somatization in both pathogenesis and maintenance of anorexia nervosa (AN), few studies have investigated the prevalence of psychosomatic syndromes in AN. The aim of this study was to use the Diagnostic Criteria for Psychosomatic Research (DCPR) to assess psychosomatic syndromes in AN and to evaluate if psychosomatic syndromes could identify subgroups of AN patients.

**Methods:**

108 AN inpatients (76 AN restricting subtype, AN-R, and 32 AN binge-purging subtype, AN-BP) were consecutively recruited and psychosomatic syndromes were diagnosed with the Structured Interview for DCPR. Participants were asked to complete psychometric tests: Body Shape Questionnaire, Beck Depression Inventory, Eating Disorder Inventory–2, and Temperament and Character Inventory. Data were submitted to cluster analysis.

**Results:**

Illness denial (63%) and alexithymia (54.6%) resulted to be the most common syndromes in our sample. Cluster analysis identified three groups: moderate psychosomatic group (49%), somatization group (26%), and severe psychosomatic group (25%). The first group was mainly represented by AN-R patients reporting often only illness denial and alexithymia as DCPR syndromes. The second group showed more severe eating and depressive symptomatology and frequently DCPR syndromes of the somatization cluster. Thanatophobia DCPR syndrome was also represented in this group. The third group reported longer duration of illness and DCPR syndromes were highly represented; in particular, all patients were found to show the alexithymia DCPR syndrome.

**Conclusions:**

These results highlight the need of a deep assessment of psychosomatic syndromes in AN. Psychosomatic syndromes correlated differently with both severity of eating symptomatology and duration of illness: therefore, DCPR could be effective to achieve tailored treatments.

## Background

It is known in scientific literature that anorexia nervosa (AN) is a psychosomatic condition [1-3]. Some authors have recently highlighted the role of several factors like alexithymia [4], emotion and anger dyscontrol [5], and somatization [6] in both development and maintenance of the disorder. It has also been described a psychosomatic and somatopsychic vicious circle as underpinning AN symptomatology [7-9].

Moreover, poor interoceptive awareness [10,11] – the inability of discerning the difference between somatic and emotional perception (i.e. feeling “fat” versus feeling angry) - is a hallmark of AN and has been recently supported by preliminary neuroscientific evidence [12].

In spite of this body of literature, there is a lack of research systematically investigating the comorbidity of psychosomatic conditions in AN [13].

The Diagnostic Criteria for Psychosomatic Research (DCPR) [14] have been proposed to deepen psychosomatic aspects. Such criteria suggest a new diagnostic frame for psychosomatic syndromes characterizing medical and psychiatric conditions to overcome the limits of the Diagnostic and Statistical Manual-IV (DSM-IV) [15] as regards somatoform disorders and to formulate a new approach for the DSM-V [14,16,17]. The DSM-IV chapter on somatoform disorders has been widely criticized [18]. In particular, its failure to adequately cover the clinical phenomena of somatisation and models of psychosomatic syndromes has been brought into question. Some authors [18,19] have acknowledged that the classification of somatoform disorders is not well supported in scientific literature and that it should be widely modified. The DSM-IV [15] shows the category of the psychological factors affecting medical conditions trying to better specify the psychological aspects but it is used in a very unspecific way. This may depend on a difficulty of a categorical psychiatric instrument, as the DSM, to recognize the subthreshold aspects of psychological distress. The DCPR system offers an alternative to DSM–IV’s somatoform disorders but also allows the clinician to characterize a patient’s mode of perceiving, recognizing, labelling, and responding to a health status [20]. DCPR variables may occur in conjunction with any psychiatric disorder listed in the DSM–IV or with any medical disorder [18].

The DCPR have been found to be more suitable than DSM-IV [15] criteria in describing psychological distress in a variety of medical settings; moreover, the DPCR represent an instrument to get through the dichotomy organic/functional. They also provide a biopsychosocial perspective in the most common medical conditions [14,21,22].

To date, the DPCR have been used in patients affected by gastrointestinal, cardiac, dermatological, endocrine, and neoplastic disorders; in addition, also in patients affected by somatoform disorder and in consultation-liaison psychiatry [20,23-29]. In a study conducted on 347 individuals from the general population, alexithymia, type A behavior, and irritable mood were found as relatively common DCPR syndromes correlating with worse quality of life whilst demoralization and persistent somatization were common in those medically ill and not in the general population [30].

More recently, the use of the DCPR allowed to individuate in a large sample of medically ill patients the association between anxiety and psychosomatic disorders [31] and psychosomatic subtypes of major depressive disorder [32].

Studies of consultation-liaison psychiatry demonstrated that about 85% of patients showed some DCPR syndromes while 51% was found to have several associated DCPR syndromes [33].

To the best of our knowledge, in literature there is only one study, conducted by our group, that applied the four syndromes of the area “Psychological factors influencing vulnerability to illness” of DPCR to 101 Eating Disorder (ED) outpatients [13]. Since it was a preliminary study we considered only the four syndromes providing a better specification of the DSM-IV rubric of psychological factors affecting medical condition, excluding the eight factors related to somatisation. Our previous paper investigated all ED diagnoses (AN restricting subtype [AN-R] and binge-purging subtype [AN-BP], Bulimia nervosa, Eating Disorder Not Otherwise Specified including Binge Eating Disorder) using the DCPR. Alexithymia was the most represented syndrome (52% of the sample), followed by demoralization (48%), irritable mood (40%), and type A behavior (27%) with the majority of the sample reporting at least one DCPR syndrome [13]. Among diagnostic categories, AN-R patients were found to show the highest frequency of psychosomatic syndromes according to the DCPR.

The aforementioned paper by Fassino and Coworkers [13] provided preliminary data on the utility of assessing psychosomatic syndromes in ED using the DCPR. It confirmed the prevalence of alexithymia, demoralization, and anger as assessed not only using psychometric measures [4,34] but also as full syndromes clinically evaluated. The clinical identification of psychosomatic syndromes confirms the theoretical psychosomatic model. The latter considers psychosomatic factors as key-aspects in the pathogenesis of ED given the central role of lived corporeality and alienation from one own’s body and one own’s emotion [35,36].

To bridge the gap between psychosomatic factors and ED we conducted this study aiming to: a) replicate and widen previous data on the prevalence of psychosomatic syndromes on AN inpatients applying all twelve criteria on a large sample; b) divide the wide and heterogeneous AN group according to the DCPR syndromes and clinical variables using a cluster analysis.

Our a priori hypothesis consisted in the identification of clusters with different prevalence of DCPR syndromes and clinical and psychosocial features.

## Methods

The study population consisted of 108 AN inpatients consecutively hospitalized at the ED Centre of the University of Turin, Italy, over a period of two years. Hospitalization occurred during patients’ emergency phase; according to international guidelines [37], hospitalization was needed on the bases of psychiatric and behavioral factors, including a rapid or persistent decline in oral intake or co-occurring psychiatric problems leading to medical instability, abnormalities in vital signs and laboratory tests. Moreover, patients’ degree of denial and resistance was high. Patients were approached by a researcher not actively participating in the clinical work.

Therefore, hospitalization was required to treat acute episodes and to provide tailored aftercare programs, including partial hospitalization. The duration of the intensive inpatient treatment was individualized and - differently from rehabilitation programs - hospitalization did not aim to achieve full weight restoration. All individuals were diagnosed with an ED, distributed as follows: 76 with AN-R and 32 with AN-BP. Exclusion criteria were: (1) medical comorbidity preceding the AN onset (e.g., epilepsy or diabetes); (2) male gender; (3) actual psychosis and drug dependence. Inclusion criteria were: (1) full-criteria diagnosis of AN [15]; (2) female gender. All participants provided written informed consent according to the Ethical Committee of the Department of Neuroscience of the University of Turin.

The initial sample included 120 individuals, but 2 patients refused to be included in this study, 6 patients were male, 3 showed severe medical comorbidity and 1 was excluded because of current psychosis (paranoid brief episode in a patient affected by severe borderline personality disorder), so the sample consisted of 108 participants.

All the patients were assessed by psychiatrists with the Structured Clinical Interview for DSM-IV Axis-I Disorders (SCID-I) [38].

All participants were asked to complete psychometric tests: Body Shape Questionnaire (BSQ) to evaluate body image, Beck Depression Inventory (BDI) for a dimensional assessment of depressive symptoms, Eating Disorder Inventory–2 (EDI-2) to assess eating symptomatology, and Temperament and Character Inventory (TCI) to dimensionally assess personality. Interviews and tests have been collected during the first week of hospitalization.

Psychosomatic syndromes were diagnosed with the Structured Interview for DCPR [39].

### The body shape questionnaire

The BSQ [40] is a 34-item self-report questionnaire that assesses body image and concerns about body shape. The participants have to respond to items regarding how they have felt about their body shape in the last few weeks choosing among 6 degrees of severity.

The BSQ showed good internal consistency (Cronbach alpha 0.97) [41] and test-retest reliability (0.88) [42].

### The beck depression inventory

The BDI [43] is a 13-item self-report questionnaire used to evaluate the severity of depressive symptoms.

The BDI demonstrated high internal consistency, with alpha coefficients of 0.86 and 0.81 for psychiatric and non-psychiatric populations, respectively [44]; Beck and Coworkers [43] did not recommend conventional test-retest reliability, however Groth-Marnat [45] reported that test retest reliabilities ranged from 0.48 to 0.86.

### The eating disorder inventory – 2

The EDI-2 [46] is a self-report inventory that measures disordered eating attitudes and behaviors and personality traits common to individuals diagnosed with ED.

The test-retest reliabilities on patients with eating disorders were between 0.81 and 0.89 and on patients with other diagnoses, revealed similar correlations, ranging from a minimum of 0.75 to a maximum of 0.94. No difference in test–retest reliability was found between the two groups of patients with eating disorders and other diagnoses. It was found a high level of internal consistency of these four scales, indicated by Cronbach’s alpha values between 0.82 and 0.93 [47].

### The temperament and character inventory

The TCI [48] is a 240-item self-administered questionnaire divided into 7 dimensions. Four of these dimensions assess temperament (Novelty Seeking [NS], Harm Avoidance [HA], Reward Dependence [RD] and Persistence [P]). The other 3 dimensions of the TCI assess character, defined as the overall personality traits acquired through experience (Self-directedness [SD], Cooperativeness [C], and Self-transcendence [ST]).

Cronbach alpha values for the TCI scales ranged from 0.60 to 0.85 for the temperament scales and from 0.82 to 0.87 for the character scales. Test-retest correlations ranged from 0.52 to 0.72 for temperament and from 0.52 to 0.71 for character dimentions [49].

### The diagnostic criteria for psychosomatic research

The DCPR are a diagnostic and conceptual framework consisting of both clinical syndromes and personality concepts used to translate psychosocial variables derived from Psychosomatics into operational instruments able to identify individual patients in psychosomatic and behavioural medicine [33]. A structured interview was developed to assess the presence of the 12 syndromes: health anxiety, irritable mood, demoralization, illness denial, alexithymia, type A behavior, thanatophobia, disease phobia, functional somatic symptoms secondary to a psychiatric disorder, persistent somatization, conversion syndrome, and anniversary reaction [39,50]. The DCPR syndromes can be identified using an interview composed of 58 questions with yes or no answers [50]. Their overlap rates with DSM-IV diagnoses showed that the DCPR syndromes were able to identify psychological dimensions that do not meet DSM-IV criteria [39]. The DCPR were developed also to provide clinicians with operational criteria for psychosomatic syndromes to overcome the limitations shown by the most often diagnosed disorders in medical settings [39].

The Structured Interview for DCPR showed excellent interrater reliability with kappa values ranging from 0.69 to 0.97 [51]. It showed also good correlations with dimensional instruments for the assessment of psychosocial distress such as the Toronto Alexithymia Scale [52,53], the Psychosocial Index [54], and the General Health Questionnaire [55].

#### Statistical analysis

For statistical analysis, we used the Statistical Package for Social Sciences (SPSS 13.0 Application Guide. Chicago: SPSS, Inc., 2004). Descriptive statistics were computed. Categorical data were compared using the Chi-square test, and continuous data were analysed using the *t*-test for independent samples.

Subsequently, a two-step cluster analysis was performed to gather cases into separate groups of patients with similar characteristics. The variables we considered to group participants were: DCPR syndromes, AN subtypes, Body Mass Index (BMI), duration of illness and age.

The two-step cluster method is a scalable cluster analysis algorithm designed to manage heterogeneous data sets. It can handle both continuous and categorical variables. The two steps are: 1) pre-cluster the cases into many small sub-clusters; and 2) cluster the sub-clusters resulting from pre-cluster step into the desired number of clusters. The log-likelihood distance measure was used, with participants assigned to the cluster leading to the largest likelihood.

A defined number of clusters was not defined a priori. Solution was found with the Bayesian Information Criterion.

Then we compared the groups: categorical data were compared using the Chi-square test, and continuous data were analysed using ANOVA and post hoc *t*-test for independent samples.

A level of significance of alpha < 0.05 was considered.

## Results

### Sociodemographic and clinical features of the sample

Table 1 shows sociodemographic and clinical characteristics of the sample. AN-BP individuals were characterized by older age and longer duration of illness.

**Table 1:** Sociodemographic and clinical features of the sample

	**AN-R**	**AN-BP**	**Total sample**	**Chi-square/t**	**p**
	**(N = 76)**	**(N = 32)**	**(N = 108)**		
Married (%)	12	14	13	0.041	0.839
Living alone (%)	6	10	9	0.211	0.644
Occupation (%)	22	28	24	0.240	0.624
Age (mean ± SD)	26.1 ± 9.1	30.7 ± 8.9	27.4 ± 9.2	−2.477	**0.016**
Years of education (mean ± SD)	12.9 ± 2.6	13.4 ± 3.1	13.1 ± 2.6	−0.479	0.637
Body Mass Index (mean ± SD)	14.8 ± 2.4	15.2 ± 3.3	14.9 ± 2.7	−0.865	0.392
Number of previous outpatient treatments (mean ± SD)	0.6 ± 0.8	0.7 ± 0.9	0.6 ± 0.9	−0.364	0.718
Duration of illness (years, mean ± SD)	7.8 ± 8.2	12.4 ± 8.4	9.1 ± 8.5	−2.652	**0.009**
Age of onset (years, mean ± SD)	18.3 ± 6.1	18.2 ± 5.2	18.3 ± 5.8	−0.072	0.942
Number of hospitalizations in the previous year (mean ± SD)	1.1 ± 1.7	1.92 ± 3.4	1.4 ± 2.4	−1.046	0.302
TCI					
Novelty seeking	16.3 ± 5.7	19.4 ± 6.1		−2.401	**0.018**
Harm avoidance	23.4 ± 11.3	21.5 ± 8.0		0.849	0.398
Reward dependence	14. ± 3.9	12.5 ± 4.5		1.762	0.081
Persistence	6.4 ± 9.6	5.2 ± 2.7		0.663	0.509
Self-directedness	23.8 ± 9.7	20.1 ± 7.5		1.840	0.069
Cooperativeness	31.0 ± 7.6	29.4 ± 7.3		0.999	0.320
Self-trascendence	12.6 ± 6.5	15.8 ± 5.9		2.346	**0.021**
EDI-2					
Drive for thinness	10.9 ± 7.6	10.2 ± 6.9		0.385	0.701
Bulimia	2.5 ± 3.7	6.1 ± 6.3		−3.516	**0.001**
Body dissatisfaction	12.2 ± 6.7	13.7 ± 7.7		−0.910	0.365
Ineffectiveness	9.6 ± 8.0	11.9 ± 8.2		−1.261	0.210
Perfectionism	5.3 ± 4.3	6.6 ± 4.6		−1.248	0.215
Interpersonal distrust	6.7 ± 5.4	8.1 ± 5.7		−1.135	0.259
Interoceptive awareness	10.4 ± 7.9	12.5 ± 9.1		−1.130	0.261
Maturity fears	7.4 ± 5.3	8.4 ± 6.3		−0.747	0.457
Asceticism	6.9 ± 4.7	9.7 ± 6.4		−2.332	**0.022**
Impulse regulation	5.9 ± 6.1	9.5 ± 7.4		−2.504	**0.014**
Social insecurity	7.5 ± 5.1	10.3 ± 5.6		−2.359	**0.020**
BSQ	103.1 ± 42.1	103.2 ± 48.3		−0.006	0.995
BDI	14.8 ± 7.5	17.0 ± 8.0		−1.259	0.211

Legend:

AN-R: Anorexia Nervosa Restricting type.

AN-BP: Anorexia Nervosa Binge-Purging type.

TCI: Temperament and Character Inventory.

EDI-2: Eating Disorder Inventory-2.

BSQ: Body Dissatisfaction Questionnaire.

BDI: Beck Depression Inventory.

All groups did not differ regarding sociodemographic variables.

### Psychiatric assessment

AN-BP patients reported higher scores on the TCI as regards NS and ST dimensions, on the EDI-2 scores in bulimia, asceticism, impulsiveness, and social insecurity scales, and on the BDI. BSQ scores were very similar between AN-R and AN-BP (see Table 1).

### Diagnostic criteria for psychosomatic research

The 6.4% of AN patients (6.6% AN-R; 6.1% AN-BP) showed no psychosomatic syndromes, 46.8% from 1 to 3 psychosomatic syndromes (51.3% AN-R; 36.4% AN-BP), and 46.8% of participants more than 3 psychosomatic syndromes (42.1% AN-R; 57.5% AN-BP). No differences were found between AN-R and AN-BP subgroups (Chi-Square 2.286, p < 0.319).

Illness denial (63%) and alexithymia (54.6%) were the most common DCPR syndromes; in fact, they were found in more than 50% of the whole AN sample. See Table 2 for details of DCPR syndromes.

**Table 2:** Psychosomatic diagnoses: percentages of affected patients for each diagnosis

	**AN-R**	**AN-BP**	**Total sample**	**Chi-square**	**p**
	**% (N)**	**% (N)**	**% (N)**		
Illness denial	61.8% (47)	65.6% (21)	63% (68)	0.138	0.710
Alexithymia	52.6% (40)	59.4% (19)	54.6% (59)	0.413	0.520
Demoralization	43.4% (33)	62.5% (20)	49.1% (53)	3.280	0.071
Functional symptoms	38.2% (29)	50% (16)	41.7% (45)	1.299	0.254
Type A behaviour	35.5% (27)	43.8% (14)	38% (41)	0.647	0.421
Irritable mood	30.3% (23)	50% (16)	36.1 (39)	3.802	0.051
Conversion symptom	21.1% (16)	28.1% (9)	23.1% (25)	0.633	0.426
Health anxiety	25% (19)	18.8% (6)	23.1% (25)	0.494	0.482
Anniversary reaction	7.9% (6)	18.8% (6)	11.1% (12)	2.687	0.101
Persistent somatization	9.2% (7)	12.5% (4)	10.2% (11)	0.266	0.606
Disease phobia	5.3% (4)	12.5% (4)	7.4% (8)	1.719	0.190
Thanatophobia	6.6% (5)	6.3% (2)	6.5% (7)	0.004	0.949

The two-step cluster analysis individuated three mutually exclusive groups.

1. The first group was the largest (N = 53) and it was composed by a larger number of AN-R individuals than the second group. In this group the DCPR syndromes were generally significantly less represented than in the other two groups. The most frequent syndromes were denial of illness and alexithymia (see Table 3). This cluster was labelled as moderate psychosomatic group.

**Table 3:** Psychosomatic diagnostic categories within each cluster

	**Moderate psychosomatic group**	**Somatization group**	**Severe psychosomatic group**	**Chi-square**	**p**
	**(N = 53) % (N)**	**(N = 28) % (N)**	**(N = 27) % (N)**		
AN-BP subtype	25% (8)	53% (17)	22% (7)		
AN-R subtype	57% (44)	15% (12)	27% (20)	15.58	**0.001**
Health anxiety	0% (0)	7% (2)	85% (23)	77.59	**0.001**
Disease phobia	0% (0)	14% (4)	15% (4)	8.18	**0.017**
Thanatophobia	2% (1)	21% (6)	0% (0)	13.85	**0.002**
Illness denial	52% (27)	54% (15)	93% (15)	13.88	**0.001**
Functional symptoms	15% (8)	64% (18)	70% (19)	29.74	**0.001**
Persistent somatization	6% (3)	21% (6)	7% (2)	5.16	0.076
Conversion symptom	12% (6)	60% (17)	7% (2)	29.716	**0.001**
Anniversary reaction	0% (0)	39% (11)	0% (0)	34.59	**0.001**
Type A behaviour	19% (10)	57% (16)	56% (15)	15.60	**0.001**
Irritable mood	2% (1)	54% (15)	85% (23)	57.99	**0.001**
Demoralization	19% (10)	61% (17)	96% (26)	44.12	**0.001**
Alexithymia	38% (20)	39% (11)	100% (27)	30.51	**0.001**

Legend:

AN-R: Anorexia Nervosa Restricting type.

AN-BP: Anorexia Nervosa Binge-Purging type.

As regards clinical and assessment comparisons among groups see Table 4.

2. The second cluster (N = 28) showed a higher BMI. In this group AN-BP patients were more frequently found than in the other two groups. DCPR syndromes were common in this group: in particular, it was characterized by higher levels of thanatophobia, conversion symptoms and anniversary reaction; they were significantly higher than both other groups. This cluster was labelled as somatization group (see Table 3).

**Table 4:** Comparison of clinical and assessment variables among groups

	**Moderate psychosomatic group**	**Somatization group**	**Severe psychosomatic group**	**F**	**F Sign**	**p Group 1 vs Group 2**	**p Group 2 vs Group 3**	**p Group 1 vs Group 3**
	**Group 1 (N = 53) mean ± SD**	**Group 2 (N = 28) mean ± SD**	**Group 3 (N = 27) mean ± SD**					
BMI	14.6 ± 1.9	16.1 ± 3.3	14.1 ± 2.7	4.88	**0.009**	**0.037**	**0.012**	1.000
EDI-2								
Bulimia	2.8 ± 4.7	6.3 ± 6.1	2.5 ± 2.4	5.31	**0.007**	**0.010**	**0.021**	1.000
Body								
dissatisfaction	11.12 ± 7	16.8 ± 6.7	12 ± 5.6	6.0	**0.004**	**0.003**	**0.046**	1.000
Ineffectiveness	7.5 ± 7.1	15.9 ± 8.5	11.0 ± 6.9	10.11	**0.001**	**0.001**	0.089	0.189
Perfectionism	4.6 ± 3.6	7.4 ± 4.7	6.4 ± 5.1	3.84	**0.025**	**0.030**	1.000	0.296
Interoceptive								
awareness	8.7 ± 7.3	17.8 ± 8.1	10 ± 6.8	12.27	**0.001**	**0.001**	**0.002**	1.000
Asceticism	5.9 ± 4.1	11.2 ± 6.4	8.4 ± 5.1	8.85	**0.001**	**0.001**	0.201	0.152
Impulsiveness	4.2 ± 5.1	13.8 ± 7	6.6 ± 4	24.5	**0.001**	**0.001**	**0.001**	0.251
Social insecurity	6.5 ± 4.9	12 ± 5.7	8.6 ± 4.2	9.5	**0.001**	**0.001**	0.078	0.277
BSQ	89 ± 34.2	140.6 ± 40.4	93.2 ± 42.8	8.33	**0.001**	**0.001**	**0.006**	1.000
TCI								
Self-Directedness	24.8 ± 9.9	16.6 ± 5.2	24.2 ± 8.2	8.19	**0.001**	**0.001**	**0.009**	1.000
BDI	12.3 ± 6.9	21.9 ± 6.6	14.7 ± 5.5	16.71	**0.001**	**0.001**	**0.002**	0.569
Duration of illness (years)	7.9 ± 8.8	7.8 ± 6.1	12.6 ± 9.1	3.25	**0.043**	1.000	0.108	**0.049**

Legend:

BMI: Body Mass Index.

EDI-2: Eating Disorder Inventory-2.

BSQ: Body Shape Questionnaire.

TCI: Temperament and Character Inventory.

BDI: Beck Depression Inventory.

As regards clinical and assessment comparisons among groups see Table 4.

3. In the third group (N = 27) AN-R patients were more represented when compared to the second group. DCPR syndromes in this group were extremely frequent: more than 85% of the patients showed health anxiety, illness denial, irritability, and demoralization. All patients in this group reported the alexithymia syndrome. This group was labelled as severe psychosomatic group (see Table 3).

As regards clinical and assessment comparisons among groups, the third group showed a longer duration of illness than group 1 (see Table 4).

Although there was no statistical significance, the first group showed shorter hospitalizations than both other groups (days of hospitalization: 29.3 ± 15.5 vs 32.8 ± 28.8 vs 32.6 ± 27.9; df = 2, F 0.291) with a shorter range between maximum and minimum duration (days of hospitalization: 3–90 vs 9–128 vs 12–160).

## Discussion

### DCPR and AN

This study aimed to assess on a sample of AN inpatients the comorbidity with psychosomatic syndromes using the DCPR [50].

The great majority of the sample reported a psychosomatic syndrome (93.6%) and almost half of the sample (46.8%) showed more than three comorbid psychosomatic syndromes, confirming the relevance of psychosomatic factors in ED [4,6], even higher than in medically ill patients [32]. Such a high frequency of psychosomatic syndromes can be due both to the exaggerated focusing on the body in AN [56,57] and to all those psychic alterations leading to somatic ones and vice versa. This spiral mechanism could play a key-role in both maintaining the disorder [8,9] and triggering the onset of psychosomatic syndromes, particularly in severe and acute patients like those of this study.

Analising each syndrome, it should be noted that two psychosomatic syndromes (illness denial and alexithymia) were diagnosed in more than 50% of AN patients and that they were both significantly represented in all clusters of this study. It is noteworthy that both syndromes are well-known factors involved in both maintaining the disorder and in resistance to treatment. These data are in line with literature since these two syndromes have been suggested as possible psychosomatic key-elements for some diagnostic or psychopathological factors [58,59].

Illness denial – the most frequent syndrome – represents an important factor to explain patients’ obstinate treatment refusal [60], high rates of drop-out [61], and their well-known difficulty to maintain a trusting relationship with their psychotherapist [57,60].

Alexithymia describes ED patients’ emotive inability and it is considered more trait rather than state-dependent in ED pathogenesis [62]. Eating symptomatology has been suggested to represent a strategy to avoid emotions [63] and the impairment in recognizing and expressing them could generate body symptoms as “concretized metaphors” describing a psychic equivalence between physical and psychic reality with concretized emotions as a result [35]. As Skårderud states “Concretized metaphors refer to instances where the metaphors are not experienced as indirect expressions showing something thus mediated, but they are experienced as direct and bodily revelations of a concrete reality. There is an immediate equivalence between bodily and emotional experience” [35].

Comparing descriptive data of the whole sample to those of our previous study on ED outpatients [13], the current sample differs as regards the higher frequency of type A behavior. This different result can be due to different sampling criteria: in fact, we previously included outpatients and individuals affected also by Binge Eating Disorder. It is indeed largely known that AN patients – compared to other ED individuals - behave in a more controlled and perfectionistic way [64]. Moreover, the present sample included AN inpatients in an emergency phase: it could also be hypothesized that type A behavior diagnostic category is less stable in time and more state-dependent than other categories.

The rates of psychosomatic syndromes obtained by using DCPR (see Table 2) did not differ significantly between AN subtypes. In AN-BP patients it could be also observed a higher prevalence of demoralization, even if not significant. These findings are in line with previous data in literature reporting a more frequent comorbid depressive symptomatology and anger dyscontrol in this subtype of AN [65,66].

### DCPR and clusters

The second aim of this study consisted in grouping the sample into more specific clusters to better characterize the link between psychosomatic syndromes and DCPR. Compared with limited DSM-IV ED diagnoses, clusters allow a more clearly differentiated characterization of patients subgroups on a broad range of features. Previous researches considering clusters had a clinical utility [67,68]. In our study, a cluster analysis allowed to identify specific association patterns between AN and psychosomatic syndromes. It is well-known that ED can also be subdivided according to personality features in different ways [69-71]. It is possible that different personality styles associated to ED can correspond to different psychosomatic clusters. In fact, AN patients who are mainly characterized by a perfectionistic and inhibited style are more represented by cluster 1 whilst AN-BP, more disinhibited and impulsive are mostly represented in cluster 2. Therefore, the use of psychosomatic clusters reinforces the idea of different personality styles and psychopathological variability in AN. From a clinical standpoint, to identify patients on the bases of their problems of somatization versus personality and alexithymia could mean to use different treatments, as debated below.

The TCI provided less data than expected as regards clusters mostly about temperament dimensions. However, it is of interest that the somatization group showed lower Self-directedness (SD) than other groups. Since SD quantifies the extent to which an individual is responsible, reliable, resourceful, goal-oriented, and self-confident it is noteworthy that the group showing a more immature character expresses distress through corporeality. Maybe this group has less resources to verbalize problems; therefore, therapy should address such an impairment: psychotherapy needs to consider the possible hindrance represented by the poorer expressive tools of this subgroup.

The cluster analysis individuated three clusters: moderate psychosomatic, somatization, and severe psychosomatic group.

The first group represented about a half of the sample and the psychosomatic syndromes were relatively scarcely represented (6 syndromes out of 12 were lower than 10%; see Table 4). The most represented syndromes of the first group were illness denial (52%) and alexithymia (38%) since these syndromes were highly represented also in other groups it could be raised the hypothesis that such features can be shared by AN individuals. It is noteworthy that this group seemed to be mostly represented by AN-R individuals (85%), with less severe eating symptomatology than group 2 and with shorter duration of illness than group 3. Given the cross-sectional design of this study it cannot be clarified if less psychosomatic syndromes are causes or consequences of severity of illness or duration of illness. However, it is clear that a smaller number of psychosomatic syndromes mean a better clinical condition at the moment of assessment. Still, even though the datum is not statistically significant, this group required shorter hospitalizations than the other groups.

The second group (somatization group) assembled patients with severe eating psychopathology, mostly AN-BP, well-known feature influencing negatively prognosis [72]. In spite of higher BMI, this finding is not in contradiction with a more severe psychopathology; in fact, BMI is representative of the somatic – rather than psychiatric – clinical severity [73]. Patients seemed to show higher character immaturity than the other groups: also this element indicates higher severity [66].

Moreover, in this group was found high depressive symptomatology, generally associated to the severity of eating psychopathology [66]. The majority of patients in this group reported psychosomatic problems (6 psychosomatic syndromes have been found in more than 50% of the sample; see Table 3). In particular, the clinical severity of this cluster was associated with thanatophobia (found almost exclusively in this group), somatization area (persistent somatization, conversion symptom and anniversary reaction) and type A behavior. These syndromes could be indeed considered as severity indexes of ED.

Thanatophobia can be associated to both a more severe psychiatric comorbidity and the awareness of higher risks of death due to hypokalemia, condition linked to purging behaviors [73].

Somatization has not been well deepened in the ED population, even if some studies have recently described the relationship between functional symptomatology and ED, in particular the gastroenteric functional symptoms [74,75]. From a clinical standpoint, it could be difficult to diagnose as organic or functional a gastrointestinal disease referred by ED patients; anyway it is well-known that ED patients’ body sensations – often inappropriately experienced – can influence the disease and its course [11]. Moreover, in this subgroup were also clearly represented some conversion symptoms, only sporadically discussed in AN patients [76,77]. Hence, it should be considered that for some AN patients – as in this cluster - body language can be a further sign of their clinical severity, requiring a clinical approach specifically tailored to these conversion aspects, often a neglected issue in AN treatment.

Still, the high frequency of type A behavior (54%) in this group could be strictly related to both need of control and perfectionism, core elements and severity indexes of AN [64].

The third group was extremely characterized by psychosomatic aspects (6 syndromes have been found in more than 70% of the sample; see Table 4). In particular, in this group have been found high percentages of health anxiety (85%) - in contrast with the other groups - and high percentages of psychosomatic syndromes of those psychological factors influencing illness vulnerability.

Moreover, AN individuals of this group showed a duration of illness significantly higher than the first group.

Anxiety traits and symptoms are widely known in a subgroup of AN patients and they can influence negatively their prognosis [78]. It is noteworthy that in this subgroup the DCPR health anxiety syndrome is associated to a longer duration of the illness. Health anxiety could be indeed explained by a heavier burden of disease after many years of AN.

Finally, almost all patients in this group deny illness (93%), hardly recognize and express their feelings (100%), and are frequently irritated (85%) or demoralized (96%). From a clinical standpoint, it should be noted that feelings of loss of hope and anger lower patients’ motivation, key element in ED treatments [79,80]. Future longitudinal researches should investigate if early demoralization can predict early drop-out, worse long-term outcome and consecutively entail a longer duration of ED or if it is the long duration of illness to make patients more alexithymic, irritable, and demoralized. It should be noted that this subgroup highlights that long-standing patients show more psychosomatic problems and that the latter can further strengthen the eating vicious circle [8]. Probably, AN treatments should mainly address the “give–up syndrome" that can arise considering patients' low motivation and previously failed treatments [8]. This intervention could be preliminary to the specific ED treatment and it could help not to thwart clinical efforts.

## Conclusions

This study shows several limitations. First, it is cross-sectional and so it is not possible to individuate to what extent DPCR in AN is state-independent or stable in time. Second, the presence of organic symptomatology in AN individuals could be a bias overestimating or underestimating some syndromes as persistent functional symptoms. Third, the considered sample is large but including only AN inpatients and so the results cannot be generalized to less severe patients. Still, more AN-R than AN-BP patients were included and it would be interesting to replicate DCPR evaluation also in other ED Centres and with a comparison group. From a statistical standpoint, corrective measures for the post-hoc test (e.g., the Bonferroni correction) were not used. Finally, a limitation of this study is the lack of a control group. However, comparing our data to the ones in literature on the general population [30], it should be noted that ED patients are more often affected by psychosomatic syndromes. When compared to depressed patients [81] the percentages of psychosomatic syndromes did not differ but illness denial and alexithymia were less represented in depressed patients that in AN, raising the hypothesis of different psychosomatic patterns among psychiatric diagnoses.

Considering the aforementioned limitations, this work highlights the role of psychosomatic factors – neglected topic in the last decades of ED research [9,13] - in AN. The study provides support to the clinical utilization of DCPR to assess patients with a more complex approach. Still, the identification of clusters could have relevant therapeutic implications. It is well-known in literature that those patients who are highly alexithymic receive overall more treatments – and significantly more antidepressants rather than psychotherapy - than those without these traits [82]. The cluster showing higher alexithymia represents instead a caveat as regards undergoing psychotherapy. Moreover, somatization issues in ED – as reported in cluster 2 - represent a quite new research field as demonstrated by the current dearth of studies; lived corporeality [36] should be addressed more in therapy with patients of this group. Given their common comorbidity with borderline personality traits – usually highlighting themes of somatic preoccupation and somatization disorder [83] -, AN-BP patients often report such features, which should be specifically considered in therapy.

The study reports indeed that the majority of AN patients shows psychosomatic elements and also that such elements are relevant for both patients’ assessment and treatments. This can support the introduction of a more complex clinical practice able to provide tailored treatments [9] in conjunction with the categorical diagnostic system reporting patients - eventually deceptively - as similar [31]. In particular, alexithymia and illness denial resulted to be common syndromes in AN individuals: these syndromes well describe some core factors of ED patients’ resistance to treatments [59,84] and drop-out [61].

Psychosomatic syndromes allow to identify subgroups of patients with different features; particularly, we found that 25% of the AN sample reported relevant psychosomatic problems probably associated to longer duration of illness. Clinicians should carefully consider these elements to provide more effective treatments and to achieve more compliance to therapy. Further longitudinal studies are warranted to shed light on psychosomatic syndromes and their utilization in clinical practice.

## Competing interests

The authors have no competing interests to declare.

## Authors’ contributions

A-DG designed the study and wrote the majority of the paper; DN revised the manuscript critically; NB contributed to write the paper; GC contributed to write the paper and edited the manuscript; ME revised and translated the paper; AF revised the manuscript critically; FS gave the final approval of the version to be published. All authors read and approved the final manuscript.

## Pre-publication history

The pre-publication history for this paper can be accessed here:

http://www.biomedcentral.com/1471-244X/13/14/prepub
